# Rapid high‐dose cyclophosphamide as bridging treatment for advanced therapies in multiple myeloma

**DOI:** 10.1002/jha2.1039

**Published:** 2024-10-20

**Authors:** Marcus Marable, Kaitlin Kelly, Jennifer H. Cooperrider, Andrzej Jakubowiak, Benjamin A. Derman

**Affiliations:** ^1^ Department of Medicine University of Chicago Chicago Illinois USA

**Keywords:** immunotherapy, multiple myeloma, myeloma therapy

## Abstract

Patients with relapsed/refractory multiple myeloma proceeding with chimeric antigen receptor (CAR) T‐cell therapy or bispecific antibodies (BsAb) may need bridging therapy to realize their benefits. We evaluated the efficacy and safety of rapid, peripheral, high‐dose cyclophosphamide (TurboCy) in 15 patients intending to proceed with CAR T‐cell therapy, BsAbs, or long‐term regimens. The overall response rate was 80% and the clinical benefit rate was 100% in a heavily pretreated high‐risk cohort. Cytopenias were common but no deaths occurred during bridging. All patients proceeded to their next line of intended therapy. TurboCy is an effective and safe bridging strategy.

1

Outcomes for multiply relapsed multiple myeloma (MM) are improving owing to the introduction of several novel therapies, mainly chimeric antigen receptor (CAR) T‐cell therapy and T‐cell engaging bispecific antibodies (BsAbs). By the time patients meet the indications for these therapies, especially B‐cell maturation antigen (BCMA)‐directed CAR T‐cell therapies such as idecabtagene vicleucel (ide‐cel) and ciltacabtagene autoleucel (cilta‐cel), they lack effective conventional chemotherapeutic treatment options for disease control while they await apheresis and CAR T‐cell manufacturing. The entire process may take up to 3–4 months, putting patients at risk for multiorgan damage or even death [1]. Indeed, some patients die waiting for their CAR T‐cell therapy, including 7.5% in the KarMMa‐3 and 6% in the CARTITUDE‐4 studies which offered effective bridging strategies. Those with progressive disease during bridging therapy also face more toxicities from CAR T‐cell therapy [2, 3]. While BsAbs are “off the shelf” therapies that can be readily administered, the median time to response is still 1.2 months for teclistamab (range 0.2–5.5), elranatamab (range 0.9–7.4), and talquetamab (range 0.3–6.8) [4, 5, 6]. Even with what is considered a relatively short time to response, this may be too long for patients with rapidly progressive disease.

High‐dose cytotoxic chemotherapy has long been a part of the armamentarium against highly aggressive MM, usually in the form of multiagent regimens such as VD‐PACE (bortezomib, dexamethasone, cisplatin, doxorubicin, cyclophosphamide, and etoposide), DCEP (dexamethasone, cyclophosphamide, etoposide, and cisplatin), or modified hyper‐CVAD (a proteasome inhibitor, doxorubicin, cyclophosphamide, and bortezomib) [7, 8, 9, 10]. While these regimens carry variable efficacy in heavily pretreated MM, they usually require multiple‐day hospitalizations, central venous access, cause profound pancytopenia, and may have deleterious effects on cardiac and/or renal function. All these intensive regimens have one thing in common—high‐dose cyclophosphamide—which may be the most effective constituent. It can be administered peripherally and to patients with hepatic or renal dysfunction. We report here on a rapid 24‐h bridging therapy of peripherally administered high‐dose cyclophosphamide for patients intending to proceed with CAR T‐cell therapy, BsAbs, or long‐term regimens.

This was a single‐center retrospective review of 15 patients with relapsed/refractory MM at the University of Chicago who received rapid high‐dose cyclophosphamide (TurboCy) between April 2021 and April 2024. The primary outcomes of interest were overall response rate (ORR), clinical benefit rate (defined as stable disease or better), and success rate in proceeding with the intended next line of therapy. Secondary outcomes included progression‐free survival following the next line of therapy (PFS2), overall survival (OS), and toxicities from TurboCy, with a focus on cytopenias, infections, renal dysfunction, and death from adverse events. The data that support the findings of this study are available upon reasonable request.

All patients were admitted for TurboCy, which was administered intravenously over 60 min at a target dose of 1500 mg/m^2^ every 12 h for two doses (3000 mg/m^2^ total); intravenous mesna was coadministered at the same dose as TurboCy along with continuous intravenous hydration at 83 cc/h unless there were concerns for volume overload (Table 1). Patients with a creatinine clearance <30 mL/min or per clinician discretion for comorbid conditions or low blood counts received a recommended 33% dose reduction (1000 mg/m^2^ per dose for a total of 2000 mg/m^2^). Patients on hemodialysis (HD) had HD delayed until at least 12 h after the last dose of TurboCy. All patients received peg‐filgrastim 6 mg on‐body injector after discharge from the hospital, and then were followed twice weekly in outpatient clinic until count recovery. During the period of neutropenia, patients were prescribed levofloxacin and fluconazole until neutrophil recovery along with standard acyclovir prophylaxis. Patients intending to receive CAR T‐cell therapy were typically treated during the vein‐to‐vein time between T‐cell apheresis and infusion.

**TABLE 1:** TurboCy regimen dosing and supportive care.

**Regimen/drugs**	**Dose**	**Schedule**	**Qualifiers**
**Antiemetics**			
Ondansetron	16 mg	Oral once	Day 1 and Day 2
Fosaprepitant	150 mg	IV once	Day 1
Dexamethasone	8–12 mg	Oral	12 mg on Day 1 8 mg once on Days 2 and 3
**Hydration**			
0.9% normal saline	83 mL/h	IV continuous	Begin ≥1 h before cyclophosphamide. May omit or reduce if on dialysis or concerns for volume overload
**Chemotherapy regimen**			
Cyclophosphamide	1500 mg/m^2^	IV every 12 h	Total two doses*; delay dialysis until ≥12 h after last dose
Mesna	1500 mg/m^2^ (1:1 ratio)	IV every 12 h	Total two doses*; Mixed in the same bag as cyclophosphamide
**Supportive care**			
Pegfilgrastim	6 mg	SQ once	Administer ≥24 h from last dose (or anytime after last cyclophosphamide dose if using on‐body injector)
Levofloxacin	500 mg	Oral daily	Until neutrophil count recovery. Adjust doses for renal function
Fluconazole		200 mg	Oral daily
Acyclovir		400 mg	Oral twice daily

Abbreviations: ECOG, Eastern Cooperative Oncology Group; SQ, subcutaneous.

* Thirty‐three percent dose reduction (cyclophosphamide 1000 mg/m^2^ for two doses and mesna 1000 mg/m^2^ for two doses) for the following reasons: creatinine clearance <30 mL/min or on dialysis, platelets <100,000/mm^3^, absolute neutrophil count <1000/mm^3^, and/or ECOG performance status ≥2. For poor performance status, consider dose reduction of 50% after weighing risks and benefits.

Of the 15 patients included in this analysis, the median age was 55 (range 37–73), with diverse patient representation (Table 2). The median time from diagnosis to TurboCy was 3 years (range 0.7–8.8). High‐risk cytogenetic abnormalities were identified in eight (53%) patients, with four (26%) harboring 2+ high‐risk abnormalities. Extramedullary disease was present in 10 (67%) patients. Nearly all patients had received prior bortezomib (93%), carfilzomib (87%), daratumumab (100%), and lenalidomide (100%); a majority had also received pomalidomide (67%) and low‐dose (weekly) cyclophosphamide (60%). High‐dose melphalan as part of autologous stem cell transplant (ASCT) was previously received by 60%, a prior BsAb in 20%, and prior CAR T‐cell therapy in 13%. All patients had triple‐class refractory disease and 67% had penta‐refractory disease prior to TurboCy. The main purpose of TurboCy was as bridging therapy to CAR T (10/15, 67%) or a BsAb (3/15, 20%); one patient was bridged to rescue ASCT and one to a conventional regimen. The median dose of TurboCy was 2060 mg/m^2^ (seven received ∼3000 mg/m^2^, seven received ∼2000 mg/m^2^ due to renal dysfunction (*n* = 6) or lower performance status (*n* = 1), and 1 received 1500 mg/m^2^ due to long‐standing cytopenias). A total of eight (53%) patients received more than one cycle of TurboCy.

**TABLE 2:** Patient and disease characteristics.

Characteristics	Patients (*N* = 15)
Median age at diagnosis	55 [37–73]
Sex	
Male	8 (53%)
Female	7 (47%)
Race	
White	7 (47%)
Black/African American	7 (47%)
Other	1 (6%)
1 HRCA/2+ HRCA	8 (53%)/4 (29%)
Extramedullary disease	10 (67%)
Yes from diagnosis, median (range)	3 (0.7–8.8)
Prior drug exposure	
Bortezomib/carfilzomib	14 (93%)/13 (87%
Lenalidomide/pomalidomide	15 (100%)/10 (67%)
Daratumumab	15 (100%)
Low‐dose cyclophosphamide	9 (60%)
ASCT	11 (73%)
BCMA CAR T‐cell therapy	2 (13%)
Bispecific antibody	3 (20%)
Triple‐class refractory disease	15 (100%)
Penta‐refractory disease	10 (67%)
Bridged to	
CAR T‐cell therapy	10 (67%)
Bispecific antibody	3 (20%)
ASCT	1 (6%)
Conventional agents	1 (6%)
Cyclophosphamide dose (mg/m^2^), median (range)	2060 (1500–3040)

Abbreviations: ASCT, autologous stem cell transplant; BCMA, B‐cell maturation antigen; CAR, chimeric antigen receptor; HRCA, high‐risk cytogenetic abnormality (17p deletion, t(4;14), t(14;16), t(14;20), or 1q amp).

The ORR was 12/15 (80%, 95% confidence interval [CI] 52%–96%) with seven (47%) patients achieving a partial response and five (33%) a very good partial response. The clinical benefit rate was 100%, with one stable disease and two minimal responses. Of these three patients with less than a partial response, two had prior cyclophosphamide exposure. All 13 patients who were intending to proceed with CAR T‐cell therapy or BsAb were able to do so. There were five deaths—three due to progression and two due to infections—which all occurred during the patients’ next line of therapy. With a median follow‐up of 7.3 months from the time of first TurboCy administration, the median OS was not reached (95% CI 6.1 months–not estimable [NE]; Figure 1A). The estimated 1‐year OS was 69% (95% CI 36%–87%). Indexing from the time of receiving the next line of therapy following TurboCy with a median follow‐up of 4.7 months, the median PFS2 was 6.0 months (95% CI 2.7–10.5; Figure 1B). Of the 10 patients bridged to CAR T‐cell therapy, the median PFS2 was 9.1 months (95% CI 2.3–NE; Figure 1C).

**FIGURE 1:**
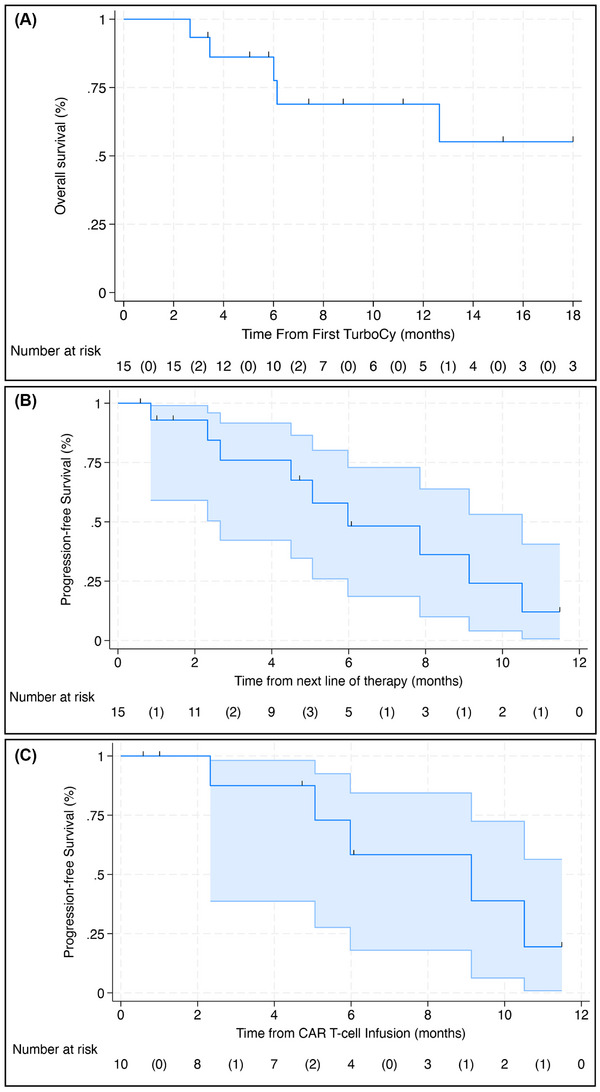
(A) Overall survival from the time of first receipt of TurboCy. (B) Progression‐free survival indexed from the time of receipt of next line of therapy (PFS2) with 95% confidence interval (CI) bands. The median PFS2 was 6.0 months (95% CI 2.7–10.5). (C) PFS2 for patients receiving chimeric antigen receptor (CAR) T‐cell therapy. The median PFS2 was 9.1 months (95% CI 2.3–NE).

Following TurboCy, cytopenias occurred in all patients. Grade 3—4 anemia occurred in 10 (67%), grade 3–4 thrombocytopenia in 10 (67%), and grade 3–4 neutropenia in nine (60%). Neutropenic fever occurred in two (13%). There were five (33%) infections, including two (13%) grade 3–4 infections. Grade 3–4 acute kidney injury occurred in two (13%), with both instances reversed quickly. No cases of hemorrhagic cystitis were observed. No patients died due to TurboCy.

TurboCy is a highly effective bridging strategy for patients with high‐risk, rapidly progressive relapsed/refractory MM and circumvents the need for prolonged hospitalization, central venous access, or organ‐threatening chemotherapy. TurboCy retained efficacy despite 60% having prior exposure to low‐dose weekly cyclophosphamide. While TurboCy did not lead directly to any patient deaths, it was not without significant toxicity. Patients receiving TurboCy must be monitored after administration for infections and bleeding complications and should receive infection prophylaxis until neutrophil recovery.

A multicenter analysis of patients receiving ide‐cel found that receipt of intensive/infusional alkylator‐based bridging therapies was associated with inferior PFS and OS compared to nonalkylator‐based therapies (median PFS 4.6 vs. 12 months, *p* = 0.002, median OS 10 months vs. not reached, *p* = 0.006) [11]. However, patients receiving intensive therapies likely had more aggressive disease biology that would be less responsive to CAR T‐cell therapy. A retrospective study evaluating hyperfractionated cyclophosphamide targeting a similar cumulative dose delivered over 4 days found an ORR of 75% but a median PFS of only 3.1 months [12]. Zafar et al. found that this hyperfractionated cyclophosphamide strategy did not lead to superior disease control compared to weekly cyclophosphamide as bridging therapy before CAR T and may have led to excess post‐CAR T toxicity [13]. However, hyperfractionated dosing over six to eight doses may have a lesser treatment effect compared to a dose‐dense strategy as done in our cohort. Retrospective studies of hyper‐CVAD, VD‐PACE, and DCEP have reported ORR of 67%, 76%, and 35%, respectively [7, 8, 10]. Patients in these cohorts all received multiagent chemotherapy over several days, which could lead to increased toxicity or have an unfavorable impact on CAR T‐cell function. In our dataset, the ORR was 80% and the mPFS following CAR T‐cell therapy was 9.1 months. Our results may compare more favorably because of better disease control with a higher dose of cyclophosphamide, and because some patients received cilta‐cel (*n* = 7) as opposed to ide‐cel (*n* = 1). The strategy of using the GPRC5D‐directed BsAb talquetamab as bridging therapy prior to BCMA‐directed CAR T‐cell therapy remains unproven, its impact on CAR T‐cell efficacy is unknown, unique toxicities still present a problem, and the time to response may not be quick enough to induce the needed response.

This study provides further evidence of the safety and efficacy of TurboCy in relapsed/refractory MM. It is familiar to many centers as it is employed as standard chemomobilization. TurboCy is a readily available, relatively safe, and effective bridging strategy for heavily pretreated MM with the additional advantages of reducing time toxicity for patients. TurboCy can be easily administered in the inpatient setting, and ongoing efforts to administer this regimen in the outpatient setting may help to further improve its convenience and utility.

## AUTHOR CONTRIBUTIONS

**Marcus Marable**: Data acquisition; data analysis; drafting of manuscript; and revision of manuscript. **Kaitlin Kelly**: Data analysis; drafting of manuscript; and revision of manuscript. **Jennifer H. Cooperrider and Andrzej Jakubowiak**: Revision of manuscript. **Benjamin A. Derman**: Study concept; data acquisition; data analysis; drafting of manuscript; and revision of manuscript. All the authors approve of the content of the manuscript.

## CONFLICT OF INTEREST STATEMENT

Marcus Marable and Kaitlin Kelly declare no personal nor financial relationships with any organization or entity with financial or nonfinancial interests in the subject matter or materials discussed in this manuscript. Jennifer H. Cooperrider declares a personal relationship with an Abbvie employee. Andrzej Jakubowiak declares honoraria and advisory board fees from Abbvie, Amgen, Bristol‐Myers Squibb/Celgene, GlaxoSmithKline, Gracell, Janssen, and Sanofi. Benjamin A. Derman declares consultancy for Johnson & Johnson, Sanofi, Canopy, and COTA; independent trial reviewer for BMS; research funding from Amgen and GSK.

## ETHICS STATEMENT

The authors have confirmed ethical approval statement is not needed for this submission.

## PATIENT CONSENT STATEMENT

The authors have confirmed patient consent statement is not needed for this submission.

## CLINICAL TRIAL REGISTRATION

The authors have confirmed clinical trial registration is not needed for this submission.

## Data Availability

The datasets generated during and/or analyzed during the current study are available from the corresponding author on reasonable request.
